# Losing Your Touch? Sustained Inattentional Numbness for Dynamic Tactile Events

**DOI:** 10.1037/xhp0001092

**Published:** 2023-04

**Authors:** Sandra Murphy, Polly Dalton

**Affiliations:** 1 Department of Psychology, Royal Holloway, University of London

**Keywords:** inattention, inattentional numbness, tactile awareness, selective attention

## Abstract

It is now well-known that a lack of attention can leave people unaware of clearly-noticeable, long-lasting and dynamic stimuli, such as a visible person dressed as a gorilla or an audible person claiming to be a gorilla. However, the question of whether touch can ever be susceptible to such extreme inattentional effects remains open. Here, we present evidence across two experiments that the absence of attention can leave people “numb” to the presence of a tactile stimulus that lasts for 3.5 s and moves across six different skin locations, establishing the new phenomenon of “sustained inattentional numbness.” The effect is particularly surprising in light of claims that tactile information processing is more direct than auditory or visual processing, which would suggest that tactile awareness might not be open to attentional modulation of the type that we observe here. The findings also have important applied implications given the increasing prevalence of tactile warnings in everyday information delivery systems.

## Public Significance Statement

This study demonstrates that people can miss a sequence of six touches moving across their skin when they are paying attention to something else. This is an important finding given the increasing prevalence of tactile warnings in everyday information delivery systems (e.g., for lane departure warnings in cars), because it suggests that these warnings may not always be noticed if they fall outside a person’s focus of attention.

Touch is a fundamental component of human experience. Its presence can strengthen all kinds of human interaction (Gallace & Spence, 2010) and its absence can lead to a range of serious problems (Ardiel & Rankin, 2010). Tactile information can also be critically important, in alerting us to stimuli that have already made contact with the body (e.g., a poisonous spider landing on the skin). Under these circumstances, we rely on attentional capture to bring unexpected tactile stimuli into our awareness when our attention has been focused elsewhere. The high potential importance of tactile stimuli makes this process of attentional capture particularly crucial in the tactile domain. However, tactile attentional capture has received relatively little research interest to date by comparison with the phenomenon as it occurs within vision and hearing.

One influential approach to investigating attentional capture has been the inattention paradigm, which has demonstrated in both vision and hearing that a lack of attention can leave people unaware of clearly-noticeable, long-lasting and dynamic stimuli, such as a visible person dressed as a gorilla (Simons & Chabris, 1999) or an audible person claiming to be a gorilla (Dalton & Fraenkel, 2012). In a typical study, participants are asked to focus on a subset of stimuli (e.g., basketball players wearing white T-shirts) while ignoring another subset (e.g., basketball players wearing black T-shirts). Once they are involved in this task, an unexpected “critical” stimulus falling into the unattended subset (e.g., a person wearing a black gorilla costume) is presented. After the critical stimulus has disappeared, observers are asked whether they noticed it, and the typical finding is that many of them have not.

One key strength of this paradigm is that it provides a direct examination of the link between attention and awareness, because participants are asked explicitly about their awareness of the critical stimulus. This is in contrast to other attentional capture paradigms, which rely on implicit measures of the extent to which the critical stimulus is processed (e.g., by examining whether the stimulus elicits cuing effects (e.g., Folk et al., 1992; Santangelo & Spence, 2007; Spence & McGlone, 2001) or whether its presence (vs. absence) slows responding (e.g., Dalton & Lavie, 2004; Theeuwes, 1992). In addition, the “one-shot” nature of the inattention paradigm allows investigation of people’s awareness of stimuli that are completely unexpected and therefore genuinely unattended—a situation that more closely approximates real-world situations than paradigms in which distractors are presented on a high proportion of the trials (e.g., Murphy & Dalton, 2016). This focus on awareness of genuinely unexpected stimuli is also crucial for understanding responses to critical real-world stimuli such as alarms, which often occur relatively rarely and without prior warning. Perhaps for these reasons, observations of sustained inattention effects in vision and hearing have been very influential, both in the attention literature and beyond. Here we ask whether similarly extreme effects can be observed within the tactile domain.

It is by no means obvious that tactile awareness will be as susceptible as visual and auditory awareness to inattention effects, particularly given the high information value that tactile stimuli can provide. Hearing and vision typically provide advance warning of approaching threats while they are still at some distance from the body; in contrast, by the time an event is signalled by the sense of touch, the relevant object will usually have made contact with the skin. For example, we are likely to hear a mosquito before we see it, and we can then use both vision and hearing to locate it and avoid it before we feel it land on our skin and start to bite. If, on the other hand, the first information we receive about the mosquito comes from the sense of touch, then an extremely quick response will be required in order to avoid being bitten. In other words, if we miss an important auditory or visual stimulus we are likely to have missed a warning of imminent danger, whereas if we miss an important tactile stimulus we may well have failed to detect the threatening event itself, with potentially dire consequences (thinking, for example, of a poisonous spider or malarial mosquito). This high survival value of tactile information is arguably reflected in its neural signalling; tactile processing has been described as being more “primitive” than auditory or visual processing because tactile sensory input is directly informative about the external environment, whereas information from other senses requires significant further decoding (Gregory, 1967). Based on these observations, it seems possible that the high survival value of tactile information will leave tactile awareness less open to attentional modulation than the other senses.

Nevertheless, some initial observations of inattentional numbness for briefly presented events have been reported. Mack and Rock (1998) asked participants to identify letters drawn (out of sight) on one of their forearms. Following several trials of this task came a “critical trial” in which a droplet of water (the “critical stimulus”) was applied without warning to the unattended forearm while the target letter was drawn on the other arm. 60% of people failed to detect this unexpected water droplet in the absence of attention. In a final control trial the participants were presented with the same stimuli as on the critical trial, but were now asked to ignore the target letter and report anything else that they felt. 87% reported the water droplet under these conditions, indicating that the stimulus was clearly detectable in full attention. Similar results emerged from an additional experiment using an air puff as the critical stimulus, and our lab has recently replicated the findings using more precisely controlled electronically-generated stimuli, with a 20 ms vibration as the critical stimulus (Murphy & Dalton, 2018). Thus the absence of attention can leave people open to “inattentional numbness,” whereby they miss tactile stimuli that are otherwise clearly noticeable.

However, the critical stimuli used in this previous research were weak, brief and static, in the sense that they involved only a single stimulus presented to one location on the skin. Here we ask whether people can also miss longer-lasting tactile events that are much more dynamic (in the sense that they involve sequences of stimulation occurring at several different skin locations). This question is theoretically important in testing the bounds of the limited tactile inattention effects that have so far been observed in the literature. The study also has practical importance given the increasing prevalence of tactile warnings in everyday information delivery systems.

We modelled our experimental design on the sustained inattention paradigm as it has been implemented within the visual and auditory domains. These studies typically involve a focal task that requires participants to focus attention on a subset of stimuli (for example, the basketball players wearing white, rather than black) so that the critical stimulus can then be presented within the unattended set (in this example, a person wearing a black gorilla suit). In order to encourage this type of strong focusing of attention within the present study, the attended and unattended stimuli were distinguished in terms of body location, such that our focal task required participants to attend to tactile stimuli on the left hand (while ignoring stimuli on the right hand) and our critical stimulus was presented to the (unattended) right arm. More specifically, for the focal task, participants made texture judgements in response to stimuli presented to their left hands while ignoring distractor textures that were simultaneously presented to their right hands. After several trials of this task, an unexpected sequence of completely different tactile stimuli, spread out across around 3.5 s and covering six different locations, was presented without warning to the right (unattended) arm by means of the activation of six electronically-controlled “tappers.” Our central question was whether the absence of attention could cause people to miss this longer-lasting dynamic tactile stimulus, as has previously been shown in the visual and auditory domains.

As explained above, our expectation that tactile awareness might be less open to attentional modulation than awareness in other sensory modalities was based partly upon the observation that tactile stimuli are likely to possess a higher survival value than auditory or visual ones. For this reason, we also attempted to manipulate the threat level of the critical tactile stimulus, with the prediction that a more threatening stimulus (in this case, one that was designed to emulate a spider-like animal crawling along the arm) should be more likely to capture attention and thus be detected regardless of the current focus of attention (Lipp & Waters, 2007). In the higher-threat “insect” condition, the tappers activated sequentially along the skin, following a pattern that was intended to suggest an insect moving closer towards the body; in the lower-threat “random” condition, the tappers activated following a random pattern.

## Experiment 1

### Methods

#### Transparency and Openness

The experiments reported here were not pre-registered. This article contains full details of sample size determination, all data exclusions, all manipulations, and all measures in the study. The experimental protocols involved manual stimulus delivery and bespoke electronics, so it is not possible to share materials. The data underlying both experiments are archived at the following link: https://osf.io/vqxzn.

#### Participants

60 people (34 female, 26 male) aged between 18 and 39 (with an average age of 20) were recruited through a departmental research participation database and gave informed consent before participating. The sample size was set in line with other published inattention studies (e.g., Dalton & Fraenkel, 2012, used 45 and 50 participants in their two inattentional deafness experiments). Importantly, though, in contrast to many earlier studies, the main research question of the current study was simply whether inattentional numbness would be seen at all for a longer-lasting dynamic tactile stimulus. This is addressed by observation and not through the use of confirmatory hypothesis testing. The secondary hypothesis (that the higher threat “insect” stimulus will be more likely to capture attention than the “random” stimulus) does require a confirmatory χ^2^ test, however because the current work describes a new experimental paradigm, there are no existing comparable data upon which to estimate the likely effect size. A sensitivity analysis (for a test of difference between two independent proportions) indicated that, for a one-tailed test with an alpha level of 0.05, and assuming a baseline detection rate of 50% in the “random” group, 40 participants (20 in each group) would allow us to detect with a power of 0.8 an effect that involved detection increasing to 86% in the “insect” group. Note that much larger effect sizes have been seen in previous work on inattentional numbness (e.g., in Murphy & Dalton, 2018, Experiment 1, we saw detection rates of 31% in the “hard” task condition rise to 92% in the “easy” task condition), however any such comparisons must be made with caution given that the paradigm presented in the current study is brand new and thus not directly comparable even to previous inattentional numbness work. Nevertheless, in the light of all these considerations, we considered 60 participants (30 per group) to be a reasonable sample size for the current work, particularly given that our main research question did not require the use of confirmatory hypothesis testing.

#### Apparatus and Stimuli

The experiment was programmed and run using Matlab and Psychtoolbox (Brainard, 1997). Participants were seated in front of a custom-built box with two cut-out holes through which they put their arms so that they remained out of sight. The experimenter was seated on the opposite side of the box, delivering the texture stimuli and recording some aspects of the participants’ responding using two foot pedals that were on the floor on the experimenter’s side of the set-up. A 15″ monitor was attached inside the box displaying visual cues to the experimenter to ensure accurate timings. All instructions to participants were delivered over headphones and spoken in a male voice, and white noise was presented during the administration of tactile stimuli to mask potential auditory cues.

We used two types of tactile stimuli: texture stimuli delivered manually by the experimenter were used for the focal task of texture change detection; brief taps delivered by electronically-controlled tappers were used for the critical stimulus.

**Figure 1 fig1:**
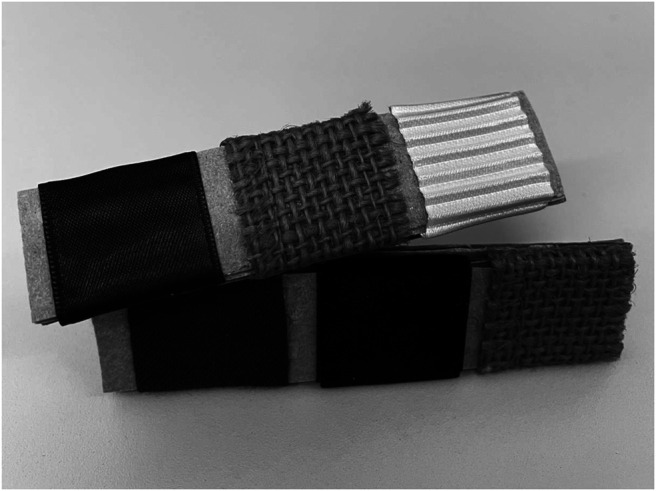
Close-Up Photograph of Two of the Tactile Texture Stimuli, Each Including Three Different Textures *Note.* Top Stimulus, Left to Right: Satin, Weave, Ridged; Bottom Stimulus, Left to Right: Linen, Velvet, Weave. Image by Polly Dalton.

For the focal texture change detection task, three pieces of fabric approximately 2.5 × 2 cm were attached to 9 × 2 cm pieces of cardboard, with a 5 mm separation between each piece of fabric. Five different fabric textures were used (linen, ridged, satin, velvet, weave) and each stimulus contained three different fabrics (see Figure 1). The back of each piece of cardboard had a short handle so that the experimenter could administer the texture to participants’ fingers in a controlled manner (see Cinel et al., 2002, for a similar approach to creating and delivering tactile texture stimuli). The tactile textures were presented for nine seconds in total, with the speed and timing of the movement controlled by the experimenter with reference to an onscreen presentation of a circle which moved at a velocity of 1 cm/s. The trial order was set such that every participant received the same stimuli for each trial. The same experimenter tested all participants and was trained to apply approximately the same force at the same speed to both hands when delivering the textures. There will of course have been some variation in the stimulus delivery given the manual approach to stimulus presentation that was necessary for this aspect of the experiment. However, the purpose of this texture task was only to focus participants’ attention on the left hand, so that the (electronically-controlled) critical stimulus could be delivered to the unattended right arm. Thus any lack of precision in presentation of the texture stimuli would not have affected the critical experimental measure (i.e., response to the critical stimulus).[Fig fig1]

**Figure 2 fig2:**
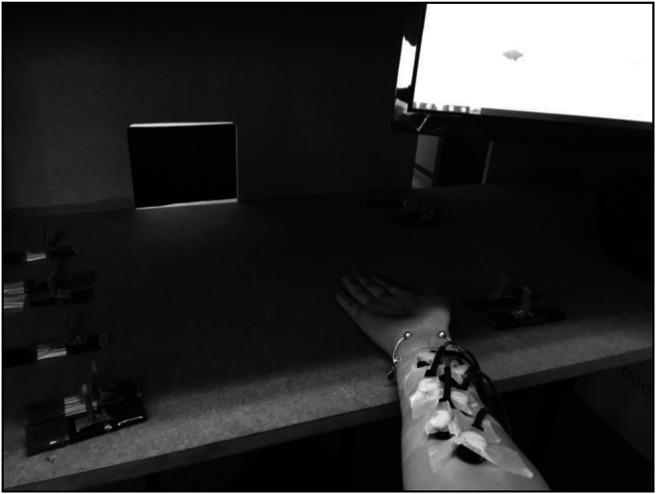
Photograph Showing Experimental Set-Up Including Screen for Guiding Experimenter Timings (Top Right), Tactile Texture Stimuli (Resting on Table) and the Experimenter’s Arm with the Tappers Attached *Note*. Although for ease of demonstration this photograph shows the tappers located on the experimenter’s arm, participants in fact sat behind this set-up (chair out of sight) and placed both their arms through the rectangular openings that can be seen at the back. Arm belongs to Sandra Murphy. Image by Polly Dalton.

The critical stimulus was presented to the right forearm using six tapper coils (http://www.psyal.com/index.php?c=104), each of which was 13.5 mm in diameter, containing a cylindrical magnet that depresses the skin when a current is run through it. As shown in Figure 2, the tappers were arranged in a zigzag pattern along the right forearm starting 5 cm from the wrist crease and held in place with medical tape. The critical stimulus consisted of a set of taps presented at 1.5 V. Each tap lasted for 30 ms, with the first, third and fifth taps followed by 670 ms inter-stimulus intervals (ISI), the second tap followed by a 770 ms ISI and the fourth tap followed by a 570 ms ISI, such that the full sequence lasted for 3,530 ms. The sequence started 3,250 ms after the onset of the nine-second presentation of the textures, meaning that the two sets of stimuli were presented concurrently. In the “insect” condition, the tappers were activated in spatial sequence, moving along the arm from the wrist towards the body. In the “random” condition, the tappers were activated in the order 6, 4, 1, 5, 2, 3 (with 1 being the location closest to the wrist and 6 being the location closest to the body).[Fig fig2]

To avoid any suspicion as to why the tappers were attached to the arm, a dummy tactile discrimination task using the tappers was performed at the very start of the experiment, prior to the onset of the task of interest. Participants were informed that this task would be alternated with the texture task, in order to provide a justification for the presence of the tappers on their arm throughout. For this dummy task, one of the six tappers delivered a stimulus that comprised either two 80 ms taps with a 10 ms off time between them (the “constant” stimulus) or a sequence of six 20 ms taps with a 10 ms off time between each (the “pulsed” stimulus). Both stimuli were presented at an intensity corresponding to the 4 V selection on the delivery apparatus. The stimulus location as well as stimulus type was fully counterbalanced.

#### Procedure

The experiments were run in 2017–2018 under the jurisdiction of the ethics committee at Royal Holloway, University of London.

Participants first performed a dummy tactile discrimination task, reporting on every trial whether the vibration delivered was constant or pulsed. 1,100 ms after stimulus onset, the spoken question “constant or pulsed?” was delivered over headphones. Participants called out their answer, which the experimenter then recorded, and feedback followed in the form of a beep over the headphones on incorrect trials. An example of each stimulus type was presented to participants, followed by 12 practice trials before a block of 48 experimental trials.

The critical phase of the experiment consisted of seven trials of the texture change detection task, followed by a “critical trial” in which the critical stimulus was unexpectedly presented along with the texture stimuli, as well as a final “full attention control trial.” In the texture change detection task, participants were presented with textures to their index, middle, and ring fingers of both hands, and were asked to focus only on the left hand. Guided by timing prompts onscreen, the experimenter delivered the textures in three continuous movements, lasting nine seconds in total (starting from the proximal digital crease towards the distal digital crease, back towards the proximal digital crease and then back towards the distal digital crease). After a five second delay (which was displayed as a countdown onscreen to ensure accurate timings), participants were presented with a second set of textures, and asked to report whether the second set was same or different to the first set (on the left hand). Following seven trials of this task, on the eighth “critical trial,” the critical stimulus was presented on the unattended right arm through activation of the tappers, one by one, starting 3,250 ms after the onset of the concurrent presentation of the textures. Immediately afterwards, participants were asked if they had felt anything unusual. If they responded yes, they were prompted to describe what they noticed. If they responded no, or if their description was incorrect or unclear, the final trial (including both the texture stimuli and the critical stimulus) was repeated with the instruction of ignoring the texture task. This final “full attention control trial” was included to ensure that the participant could detect the critical stimulus when their attention was not focused elsewhere, to rule out the possibility that the stimulus may have been simply too weak or brief, or too strongly masked by the concurrent texture stimuli (e.g., Craig, 1974; D’Amour & Harris, 2013), to be noticed at all and thus to ensure that any observed failures of awareness could be attributed to the effects of inattention rather than to lower-level perceptual factors.

### Results and Discussion

Four people were excluded from the analyses, three due to technical errors by the experimenter and one due to a below-chance score in the initial dummy tactile discrimination task (suggestive of poor overall tactile sensitivity). All of these exclusions were from the “insect” condition, leaving 26 participants in the analysis for that condition.

The results of central interest concern whether or not participants noticed the critical stimulus on the “critical” and “control” trials. Participants were classified as “missed” if they failed to notice the critical stimulus when it was first presented during the texture discrimination task, but identified it correctly in the subsequent full attention control trial. This pattern of responding indicates that the participant was clearly capable of detecting the stimulus, but failed to notice it when paying attention elsewhere, hence experiencing “inattentional numbness.” Participants who correctly reported the critical stimulus on first presentation were classified as “noticed,” and those who failed to notice it even on the final full attention control trial were classified as “failed control.” Table 1 shows the response classifications from both of the experiments in this paper.[Table tbl1]

In Experiment 1, no significant difference was observed between the patterns of awareness demonstrated by participants in the “insect” and “random” conditions of Experiment 1 (χ^2^[2, *N* = 56] = 1.63, *p* = .44). It is possible that a stimulus that was more “insect-like” (e.g., faster moving and/or covering more skin locations) may have been more effective in capturing attention than the relatively simple “insect” stimulus that we used. However, the much more important observation is that, in both conditions, at least half of the people experienced “inattentional numbness,” such that they failed to notice a multi-tap dynamic tactile stimulus in the absence of attention, despite being able to detect the very same stimulus in the subsequent full attention control trial. This suggests that tactile awareness may indeed be open to inattentional failures that are comparable to those seen in the auditory and visual domains.

However, significant numbers of participants in Experiment 1 (more than a quarter in the “insect” condition) failed the control task, such that they could not detect the critical stimulus even under full attention. This suggests that the high levels of inattentional numbness seen in this experiment could be explained at least in part by the critical stimulus being fairly difficult to detect in general (which would not be the case, for example, for critical tactile alerts). For this reason, Experiment 2 tested people’s awareness of a more intense critical stimulus.

The texture change detection task was included only as a means of focusing participants’ attention away from the upcoming location of the critical stimulus, and thus performance on this task was not of central interest for the current purposes. Nevertheless, it is possible that performance on this task could have influenced critical stimulus detection, such that participants who were focused more intently on this task might have been less likely to detect the critical stimulus than those who were not so intently focused. In order to examine this possibility, we calculated overall performance in the texture change detection task in terms of percentage of correct answers. Performance was reasonable in general, and was similar between the two groups, with average scores of 70% correct in the “missed” group (95% CI [64%, 76%]) and 66% in the “noticed” group (95% CI [57%, 75%]).

## Experiment 2

In order to confirm that inattentional numbness could occur for an unexpected stimulus that was easier to detect under full attention than those used in Experiment 1, Experiment 2 examined people’s awareness of a more intense critical stimulus. Given that Experiment 1 had not identified a significant difference between the two critical stimuli used, the current experiment focused only on the “insect” stimulus, as this had been designed with the aim of being the most similar to the types of tactile stimuli that we encounter in everyday life.

### Methods

#### Participants

30 people (22 female, 8 male) aged between 18 and 50 (with an average age of 23) were recruited through a departmental research participation database.

#### Apparatus, Stimuli and Procedure

The stimuli were identical to those of Experiment 1 with the exception of the intensity of the tactile stimulation. In the dummy tactile discrimination task, the stimuli were now presented at an intensity corresponding to the 4.5 V selection on the delivery apparatus (as opposed to 4 V in Experiment 1) and the critical stimulus was delivered at 2.5 V (as opposed to 1.5 V in Experiment 1). The apparatus and procedure were identical to those of Experiment 1.

### Results and Discussion

One person was removed from the analyses due to a below-chance score in the dummy tactile discrimination task (suggestive of poor overall tactile sensitivity). This left 29 people in the final analysis.

As can be seen in Table 1, the detectability of the critical stimulus was successfully increased by comparison with Experiment 1, such that only two people failed the control trial (a rate of 7%, as compared with 27% for the “insect” stimulus in Experiment 1). Yet despite this increased overall detectability, nearly a third of participants still failed to notice the critical stimulus, once again indicating significant levels of inattentional numbness for a multi-tap dynamic tactile event.

Performance in the texture change detection task was similar to that of Experiment 1, and was again comparable between the “missed” group (71%; 95% CI [55%, 87%]) and the “noticed” group (67%; 95% CI [60%, 74%]). However, because the numerical trends in both experiments were for better performance in the “missed” (vs. the “noticed”) group, we combined the data from both experiments to provide a reasonable sample size for statistical comparison between the two groups (recall that, while the critical stimulus varied between the two experiments, the texture change detection task did not). In total there were 40 participants in the “missed” group (*M* = 70%; 95% CI [65%, 75%]) and 32 in the “noticed” group (*M* = 67%; 95% CI [62%, 72%]), but no significant difference was observed in texture change detection performance between the two groups (*t* < 1).

## Comparisons Between Auditory and Tactile Inattention Effects

One of our initial predictions was that the sense of touch might be less susceptible to inattentional failures than the other senses, because of the high information value that tactile sensation provides. In order to test this possibility, we sought to compare the magnitude of the inattention effects seen here with those of the effects previously demonstrated in the auditory and visual domains, in order to determine whether one modality might be more or less susceptible to inattention effects than another. This type of direct comparison is not straightforward, because it is difficult to equate the baseline detectability of critical stimuli that are presented in different modalities (particularly with the “one-shot” approach that the paradigm used in the current work requires). Nevertheless, some insight can be gained from examining the rate at which participants fail to detect each critical stimulus under full attention. For the purposes of this comparison, we focus here on Experiment 2, as this contained the most clearly detectable of the three critical stimuli that we tested.

The critical tactile stimulus used in Experiment 2 was missed under full attention by 7% of participants. By comparison, however, only 2% of the participants in Dalton and Fraenkel’s (2012) Experiment 1 failed to detect the auditory “gorilla” under full attention, suggesting that their auditory “gorilla” had slightly higher detectability than the critical tactile stimulus used here. Nevertheless, the rate of inattentional deafness observed in the “attend women” condition of Dalton and Fraenkel’s Experiment 1 (70%) was significantly higher than the rate of inattentional numbness seen in Experiment 2 of the present paper (which was 33% excluding the “failed control” participants, which was necessary in order to enable a statistical comparison between the two experiments; χ^2^(1, *N* = 47) = 6.18, *p* = .013). This suggests, in line with our initial prediction, that the inattention effect observed within the current tactile paradigm may have been weaker than that seen by Dalton and Fraenkel in their auditory paradigm.

Unfortunately, Simons and Chabris (1999) did not report a formal control trial as part of their study, meaning that a similar comparison is not possible with their visual study. But these comparisons must in any case remain speculative given the difficulties involved in matching the demands of tasks run in different modalities. For example, although we provided a plausible explanation for the presence of the tactile stimulators on the unattended arm, participants may nevertheless have maintained some level of expectation of an event happening there in a way that may not have occurred in the visual and auditory paradigms. It is also possible that the attention-focusing task of listening to the conversation in Dalton and Fraenkel’s auditory scene was more demanding (or more engaging in some other way) than the texture change detection task used in the current work, and that differences in participants’ engagement with the focal task could thus have been responsible for the differences in magnitude of the attention effects observed in the two studies. Although we measured performance in the tactile change detection task, Dalton and Fraenkel did not record performance data related to the conversation monitoring task, meaning that we are unable to make comparisons between participants’ performance in the focal task between the two studies. And even if such comparisons were possible, matching performance levels across two entirely different tasks does not necessarily indicate that the two tasks were equally demanding and/or engaging. This remains an enduring challenge for between-modality comparisons for which there are no easy answers.

## General Discussion

The current findings indicate that the absence of attention can cause people to miss a clearly detectable and dynamic tactile stimulus that is presented over 3.5 s. This work extends previous demonstrations of inattentional numbness beyond the highly artificial brief, static stimuli used in earlier research (including our own) to encompass a longer-lasting dynamic tactile event that is much closer to the types of stimulus that we are likely to encounter in real life.

These findings add to a growing body of literature demonstrating the importance of attention in determining tactile awareness. For example, research into “tactile change blindness” has demonstrated that a change introduced between two successive versions of a tactile array can often go unnoticed (e.g., Gallace et al., 2006; Geldard & Sherrick, 1965) but that change detection under these circumstances can be improved if attention is directed towards the likely location of the changing stimulus (Van Hulle et al., 2013). This failure to detect tactile changes in the absence of spatial attention can be likened to the inattentional failures that we have observed in the current experiments. However, there are two critical differences between the findings that are revealed by these different paradigms. Firstly, participants in the change detection paradigm are told that a change may occur and their search for this change is thus deliberate. In contrast, the inattention paradigm is designed to investigate people’s ability to notice one-off and completely unexpected stimuli. Secondly, change detection as it is typically implemented in the laboratory requires participants to compare successive tactile arrays, imposing significant memory requirements, whereas the inattention paradigm investigates tactile awareness within a single ongoing stimulus array.

In demonstrating clear effects of inattentional numbness for dynamic tactile stimuli, the current findings indicate that the sense of touch is open to inattentional failures that are comparable in qualitative terms to those previously demonstrated within hearing and vision—a finding that seems particularly surprising given the potentially high information value that the sense of touch conveys. However, notwithstanding the difficulties involved in making direct comparisons between tasks in different modalities, our results also hint at the intriguing possibility that the sense of touch may be somewhat less open to these types of inattentional failures than the sense of hearing.

This tentative suggestion that tactile information may be less susceptible to inattentional neglect than auditory information might be taken to support the use of tactile warnings in high stakes real-world contexts such as alarm systems. However, our findings also suggest that tactile stimuli can nevertheless be missed if attention is focused strongly elsewhere, so any introduction of tactile warnings must be implemented with careful consideration of the other demands that the user is likely to be encountering when the alarm is triggered.

## Figures and Tables

**Table 1 tbl1:** Proportions of Participants Classified as “Missed,” “Noticed” and “Failed Control” [with 95% Confidence Intervals in Square Brackets]

Condition	Missed	Noticed	Failed control
Experiment 1 (“insect”)	50% [32, 68]	23% [11, 42]	27% [14, 46]
Experiment 1 (“random”)	60% [42, 75]	27% [14, 44]	13% [5, 30]
Experiment 2 (“insect”)	31% [17, 49]	62% [44, 77]	7% [2, 22]
